# Linking loggerhead locations: using multiple methods to determine the origin of sea turtles in feeding grounds

**DOI:** 10.1007/s00227-016-3055-z

**Published:** 2017-01-13

**Authors:** ALan F. Rees, Carlos Carreras, Annette C. Broderick, Dimitris Margaritoulis, Thomas B. Stringell, Brendan J. Godley

**Affiliations:** 1 ARCHELON, the Sea Turtle Protection Society of Greece, Solomou 57, 104 32 Athens, Greece; 2 Marine Turtle Research Group, Centre for Ecology and Conservation, University of Exeter, Penryn Campus, Penryn, Cornwall TR10 9FE UK; 3 Department of Genetics, Microbiology and Statistics and IRBio, University of Barcelona, Av.Diagonal 643, 08028 Barcelona, Spain

## Abstract

Many marine megavertebrate taxa, including sea turtles, disperse widely from their hatching or birthing locations but display natal homing as adults. We used flipper tagging, satellite tracking and genetics to identify the origin of loggerhead turtles living in Amvrakikos Gulf, Greece. This location has been identified as hosting regionally important numbers of large-juvenile to adult sized turtles that display long-term residency and/or association to the area, and also presents a male biased sex ratio for adults. A total of 20 individuals were linked to nesting areas in Greece through flipper tagging and satellite telemetry, with the majority (16) associated with Zakynthos Island. One additional female was tracked from Amvrakikos Gulf to Turkey where she likely nested. Mitochondrial DNA mixed stock analyses of turtles captured in Amvrakikos Gulf (*n* = 95) indicated 82% of individuals originated from Greek nesting stocks, mainly from Zakynthos Island (63%), with lesser contributions from central Turkey, Cyprus and Libya. These results suggest that the male-biased sex ratio found in Amvrakikos Gulf may be driven by the fact that males breed twice as frequently on Zakynthos, resulting in their using foraging grounds of greater proximity to the breeding site. Conservation measures in localised foraging habitats for the protection of marine vertebrates, such as sea turtles, may have positive impacts on several disparate breeding stocks and the use of multiple methods to determine source populations can indicate the relative effectiveness of these measures.

**Electronic supplementary material:**

The online version of this article (doi:10.1007/s00227-016-3055-z) contains supplementary material, which is available to authorized users.

## Introduction

Distribution and dispersal of many marine megavertebrate taxa involve large spatial scales (e.g. Pendoley et al. 2014 and additionally; tuna: Block et al. 2005; sharks: Domeier and Nasby-Lucas 2008; albatrosses: Weimerskirch et al. 2006; seals: Field et al. 2005; whales: Zerbini et al. 2006). Understanding and quantifying the intra-species connectivity between distant habitats is important for conservation and management (Webster et al. 2002; Gerber and Heppell 2004; Kennedy et al. 2014) and may necessitate international cooperation and collaboration for effective species protection (Jodice and Suryan 2010). Tagging and satellite tracking technologies have revealed a wealth of information on marine vertebrate life histories and migratory behaviour (Cooke 2008; Block et al. 2011) and genetic studies have revealed cryptic population structures (e.g. Blower et al. 2012) that are essential for the biological delineation of management units of endangered species (Moritz 1994).

Sea turtles may traverse oceans (Bowen et al. 1995; Hays et al. 2002) during their development and maturation that may take several decades (Heppell et al. 2003). Natal homing in these species contributes to genetic structuring of breeding populations so that adult turtles from different nesting areas can be identified genetically (Amorocho et al. 2012; Leroux et al. 2012). Furthermore, the pattern of adult dispersion from breeding areas has been shown to reflect the extent of passive dispersion that would be experienced by hatchlings; this, prevailing oceanography around nesting areas may be crucial to the selection of foraging sites used by adult sea turtles (Hays et al. 2010a). As for other taxa, tagging, tracking and genetic studies of sea turtles can reveal movements of individuals and stock composition in foraging grounds. For example, foraging assemblages have been shown to comprise of mixtures of turtle stocks, from often-distant rookeries (Lahanas et al. 1998; Luke et al. 2004; Bowen et al. 2007; Blumenthal et al. 2009; Nishizawa et al. 2014a).

Breeding populations and regional management units (RMUs) for loggerhead turtles have been defined using behavioural and genetic data respectively (Bowen et al. 2004; Wallace et al. 2010) and seasonal and ontogenetic movements have been identified at several locations (Limpus and Limpus 2001; Hawkes et al. 2007; McClellan and Read 2007; Casale et al. 2008; Mansfield et al. 2009; Hays et al. 2010a; Marcovaldi et al. 2010; Rees et al. 2010; Nishizawa et al. 2014b). The recent use of more variable genetic markers and extended coverage of nesting areas has increased the resolution of mitochondrial DNA (mtDNA) studies, aiding the identification of further sub-populations (Shamblin et al. 2014). This improved baseline can contribute to increased resolution in mixed stock analyses (MSAs) of foraging aggregations, which are used to highlight likely turtle origin and movements and possible conservation requirements (Roberts et al. 2005; Carreras et al. 2006; Reece et al. 2006; Monzón-Argüello et al. 2012; Garofalo et al. 2013; LaCasella et al. 2013).

The Mediterranean Sea hosts an independent loggerhead turtle RMU but is also visited by individuals from two Atlantic RMUs (Wallace et al. 2010), with individuals from the Atlantic mainly occurring in the western Mediterranean basin (Clusa et al. 2014). Mediterranean loggerhead turtles are considered to form an isolated meta-population (Carreras et al. 2011; Clusa et al. 2013) with most nesting occurring in Greece, Turkey, Cyprus and Libya (Casale and Margaritoulis 2010). The life-history for this metapopulation has been presented in the literature; hatchlings enter oceanographic currents in which they disperse to varied locations away from the breeding area (Hays et al. 2010a; Casale and Mariani 2014). As the turtles increase in size they are capable of more directed movements and may undertake long migrations through oceanic and neritic waters (Bentivegna 2002; Casale et al. 2007; Hochscheid et al. 2010; Casale et al. 2012a) with a protracted switch from pelagic to benthic foraging (Laurent et al. 1998; Casale et al. 2008). In general, they complete maturation in locations closer to their natal origin than would be found by random dispersal (Laurent et al. 1998, Maffucci et al. 2006, Garofalo et al. 2013).

As adults, maternal philopatry to breeding areas has created several reproductive clades across the eastern Mediterranean as suggested through mtDNA analysis (Carreras et al. 2007; Garofalo et al. 2009; Yilmaz et al. 2011; Saied et al. 2012; Clusa et al. 2013), but some male mediated gene flow may reduce the overall depth of reproductive isolation (Carreras et al. 2007; Yilmaz et al. 2011). The neritic area off North Africa is an important foraging habitat away from the breeding areas, used by adult turtles from Greece (Margaritoulis et al. 2003; Zbinden et al. 2011; Schofield et al. 2013), Cyprus (Broderick et al. 2007; Snape et al. 2016) and Libya (Casale et al. 2013) with no published data for loggerhead turtles nesting in Turkey. The Adriatic Sea is also an important foraging/overwintering area for turtles that breed in Greece (Margaritoulis et al. 2003; Zbinden et al. 2011; Schofield et al. 2013; Giovannotti et al. 2010) with lower numbers recorded foraging elsewhere around the Mediterranean, including the Aegean Sea, the Levant coast and Cyprus (Margaritoulis et al. 2003; Margaritoulis and Rees 2011, Snape et al. 2016).

An important foraging aggregation of loggerhead turtles has been identified in Amvrakikos Gulf, western Greece, comprising numerous large-juvenile and adult individuals. Rees et al. (2013) revealed a male-biased sex ratio, which is at odds with established widespread female-biased hatchling production (Witt et al. 2010a). To address the general lack of knowledge on the origins of loggerhead turtles in this important foraging area, we used a combination of flipper tagging, satellite tracking and genetic investigations to: (1) Identify direct linkages between the foraging area and breeding grounds. (2) Quantify composition of the foraging aggregation of loggerhead turtle breeding stocks. (3) Determine possible reasons for the previously highlighted male-biased sex ratio present in the Gulf.

## Methods

### Flipper tagging and sample collection

**Fig. 1:**
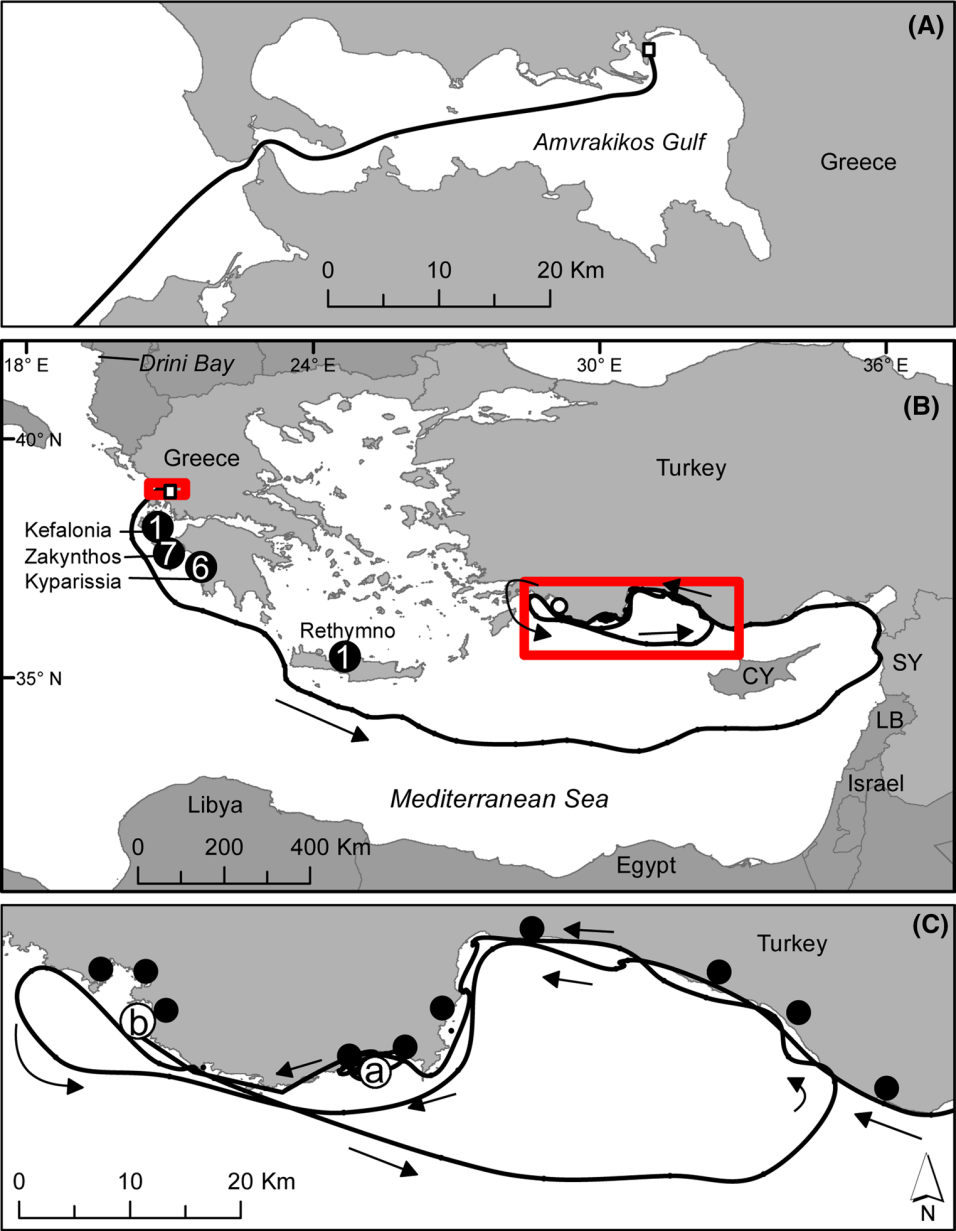
Linkage between Amvrakikos Gulf and nesting sites through satellite tracking and flipper tagging. **a** Release location and initial migration of the turtle tracked out of Amvrakikos Gulf in 2003. **b** Long-distance migration to Syria and Turkey of the same turtle.* Arrows* indicate direction of travel. *Open circle* end point. *Black circles* indicate nesting areas linked to Amvrakikos Gulf through flipper-tagged nesting individuals. *Numerals* indicate the number of turtles from the nesting site linked with Amvrakikos Gulf (*n* = 15 links from 14 individuals, as one individual nested at both Zakynthos and Kyparissia). (CY = Cyprus, LB = Lebanon, SY = Syria.). **c** *Filled circles* known loggerhead nesting areas overlapping the migratory route. *Open circles a* overwintering location (October 2003–May 2004) and b end of track (June 2004) near Fethiye nesting beach

All turtles were obtained from an extensive (Ca. 16 km^2^) shallow region (<2 m deep) in the northeast part of Amvrakikos Gulf (39°1.3′ N, 21°3.6′ E; Fig. 1), except for 2013 when a number of turtles were captured in a similarly sized area further west (39°1.6′ N, 20°57.2′ E). Annual capture intensity varied over the period 2002–2015. In 2002 and 2003, turtles were encircled with a net to capture them. In subsequent years, captures were made using turtle rodeo technique (Ehrhart and Ogren 1999) from an inflatable dinghy. All captured turtles were hauled on-board the dinghy and flipper tagged. GPS location at the capture site, curved carapace length (CCL) measurements and skin samples, from one of the rear flippers, were also taken; samples were stored in 90% ethanol until analysis. See Rees et al. (2013) for further details on methods and location of captures.

### Satellite tracking

We tracked 13 adult and juvenile turtles using Kiwisat Argos-linked satellite transmitters (Sirtrack Ltd., Havelock North, New Zealand). Three were deployed in June 2002, three in May 2003, six in June 2013 (three each from both the east and west capture locations) and one in August 2013 (from the eastern capture location). Captured turtles were taken ashore, close to the capture site, and retained in a shaded, wooden corral to affix the transmitters and were then released at the water’s edge. In 2002/2003, the fibreglass and polyester resin attachment method of Balazs et al. (1996) was employed and to extend battery life, transmitters were duty-cycled to be continuously on for the first 28 days, followed by 24 h on: 36 h off for the rest of the transmitter’s functional duration. In 2013, two-part epoxy was used to attach the transmitters to the turtles (Godley et al. 2002) and the transmitters were duty cycled to be continuously on for their entire functional duration. Location data were calculated by the Argos system (http://www.argos-system.org/). Data from the 2002/2003 transmitters were manually retrieved through telnet on a daily basis. Compiled datasets were then uploaded into the Satellite Tracking and Analysis Tool (STAT; Coyne and Godley 2005) for processing and filtering. Data from the 2013 transmitters were automatically downloaded and processed in STAT. To determine turtle routes, raw Argos data were filtered in the following manner: Location Classes 3, 2, 1, A and B were used (Witt et al. 2010b) and locations requiring excessive speed (>5 km h^−1^) or unlikely turning angle (<25°) were omitted. Locations that passed filtering were averaged to produce a single daily location.

Additionally, to complement the results of our own investigation, we undertook a review of published literature on loggerhead turtle tracking in the Mediterranean to identify any turtles recorded as migrating into Amvrakikos Gulf.

### DNA extraction and sequencing

Skin samples were processed from turtles captured in Amvrakikos Gulf between 2008 and 2011. DNA from 95 samples was extracted using the Phire^®^ Animal Tissue Direct PCR Kit (Finnzymes) and ~800 bp fragment of the mtDNA D-loop control region was amplified by polymer chain reaction (PCR) using the primer pair LCM15382 (5′-GCTTAACCCTAAGCATTGG-3′) and H950 (5′-GTCTCGGATTTAGGGGTTT-3′) (Abreu-Grobois et al. 2006). PCR protocol comprised an initial denaturation phase of 98 °C for 5 min followed by 40 cycles of 5 s at 98 °C, 5 s at 60.6 °C and 20 s at 72 °C, with a final 1 min at 72 °C before cooling to 4 °C to hold. The resulting PCR product was visualised in agarose gel before being purified by enzymatic reactions (Exo 1 and FastAP; Fermentas). Sequencing was undertaken on an Automatic Sequencer 3730XL, in both forward and reverse directions (Macrogen Europe, the Netherlands). These ~800 bp sequences were aligned in Geneious v5.5 (Drummond et al. 2011) or BioEdit v7.1.11 (Hall 1999) and compared to known loggerhead turtle haplotypes found in the database maintained by the Archie Carr Center for Sea Turtle Research (http://accstr.ufl.edu/).

### Mixed stock analysis

A ‘*one to many*’ MSA using the genetic data was conducted to test the contribution of each nesting area to the foraging area of Amvrakikos using a Bayesian approach (Pella and Masuda 2001). Hierarchical Bayesian models also allow combination of genetic and ecological data, like rookery size, to avoid the over representation of extremely small populations typical of other Bayesian approaches (Okuyama and Bolker 2005). We used the programme Bayes and applied the BM2 model from previous studies, that used the rookery size as a weighting factor, as shown to be appropriate for sea turtle studies (Bass et al. 2004). As a baseline we used published haplotype frequencies of the Atlantic and Mediterranean nesting beaches (Shamblin et al. 2014). Mean annual nests per year were also obtained from the literature as an approximation of population size (López-Jurado et al. 2007; Casale and Margaritoulis 2010; Shamblin et al. 2012). The Gelman-Rubin shrink factor was used diagnostically to test for anomalous realizations of the Bayes predictive posterior distribution (Pella and Masuda 2001) 95% CI were obtained directly from the MCMC once convergence has been reached and burn-in discarded. Values greater than 1.2 indicated a lack of convergence in the algorithm and the corresponding estimates were considered unreliable. A ‘*many to many’* mixed stock analysis (Bolker et al. 2007) was not performed as it requires the inclusion of all available foraging areas, and so far only a few feeding grounds (Yilmaz et al. 2012, Garofalo et al. 2013) have been assessed using the ~800 bp fragment.

Additionally, we performed a similar MSA using the haplotype frequencies from loggerhead turtles captured at Drini Bay in Albania (Fig. 1; Yilmaz et al. 2012) in an MSA similar to that performed on the Amvrakikos haplotype frequency results in order to compare breeding stock contributions to foraging grounds separated by approximately 350 km.

## Results

### Links from flipper tagging

In over a decade of the capture-mark-recapture study in Amvrakikos Gulf, with records of almost 700 unique individuals, identified through tag recaptures (Rees et al. 2013 and unpubl data), linkage to several nesting areas in Greece has been confirmed through flipper tag observations at Greece’s main nesting areas, which are monitored by ARCHELON. A total of ten turtles tagged at Greek nesting beaches have so far been encountered in Amvrakikos Gulf. Four turtles originally tagged nesting on Zakynthos Island (180 km from Amvrakikos) and four from southern Kyparissia Bay (240 km from Amvrakikos) comprise the majority; the remainder were a single turtle that nested at Rethymno, Crete (600 km from Amvrakikos) and a turtle tagged nesting on Kefalonia Island (130 km from Amvrakikos) that was reported to the authors by local fishers. Additionally, four turtles tagged in the Gulf were subsequently observed nesting in southern Kyparissia (1), Zakynthos (2) and both southern Kyparissia and Zakynthos during a single breeding season (1). In total 50% (7 of 14) of turtles linked to the Gulf from a nesting area were associated with Zakynthos. Details of these observations are provided in Table 1 and Fig. 1.

**Table 1:** Links to nesting areas derived from flipper tag recaptures and satellite tracking(*)

Breeding	Sex	CCL (cm)	Origin	Re-observation	Source
ZAK	F	73.5	AMV 2004	ZAK 2011. AMV 2013	Present study
ZAK	F	73.5	AMV 2005	ZAK 2010. AMV 2010, 2011	Present study
ZAK, KYP	F	73.5	AMV 2007	ZAK & KYP 2009	Present study
KYP	F	80.5	AMV 2007	KYP 2009. AMV 2012	Present study
CRE	F	78.5	CRE	AMV 2005	Present study
KEF	F	–	KEF	AMV 2002	Reported to ARCHELON
KYP	F	81.0	KYP	AMV 2002	Present study
KYP	F	81.0	KYP	AMV 2013, 2014, 2015	Present study
KYP	F	85.0	KYP	AMV 2014	Present study
ZAK	F	77.0	ZAK	AMV 2005	Present study
ZAK	F	88.5	ZAK	AMV 2005, 2007, 2011, 2015	Present study
KYP	F	83.5	KYP	AMV 2005	Present study
ZAK	F	81.5	ZAK	AMV 2007	Present study
ZAK	F	88.0	ZAK	AMV 2008, 2010	Present study
ZAK	F	86.0	ZAK 2007	AMV 2007	Zbinden et al. (2011)*
ZAK	F	76.0	ZAK 2007	AMV 2007	Zbinden et al. (2011)*
ZAK	F	83.0	ZAK 2009	AMV 2009	Schofield et al. (2013)*
ZAK	M	90.0	ZAK 2008	AMV 2008, 2009	Schofield et al. (2013)*
ZAK	M	89.0	ZAK 2009	AMV 2009	Schofield et al. (2013)*
ZAK	M	88.0	ZAK 2011	AMV 2011	Schofield et al. (2013)*

See Fig. 1 for location of nesting areas

*Breeding* breeding area where turtles were observed, *CCL* curved carapace length, *AMV* Amvrakikos Gulf, *KEF* Mounda beach—Kefalonia, *KYP* southern Kyparissia Bay, *ZAK* Laganas Bay—Zakynthos, *CRE* Rethymno—Crete, *Origin* place first tagged including year of tagging for AMV tagged and ZAK satellite tracked turtles, *Re-observation* Location and year of subsequent observation(s)

### Links from satellite telemetry

The majority (11 of 13) satellite tracked turtles remained within the gulf and the behaviours of those from 2002/2003 (*n* = 6) are discussed in Rees et al. (2013). One turtle from June 2013 (probable adult, female, CCL = 75 cm) departed the gulf on 28th June and migrated into the Adriatic Sea where it resided until 18th May 2015, when its transmitter stopped functioning. Turtle nesting does not occur in that region; hence no nesting site linkage was determined. The remaining turtle (probable adult, female, CCL = 75 cm), tagged in May 2003, departed the Gulf and its movements are presented in Fig. 1. This turtle left the Gulf on 3rd July and made an oceanic migration, reaching Syria on 14th August, with a minimum possible distance travelled from origin of 1650 km. It then moved north and westwards following a more coastal route in Turkey. After undertaking a large oceanic loop, it returned to the Turkish coast and arrived at its overwintering spot near the Kale and Kumluca nesting areas on 23rd October, having travelled a total of 3425 km. It remained there through to May 2004 until it travelled westwards, with the last location received on 24th June (mid-nesting season in the Mediterranean), 15 km from Fethiye beach, a known loggerhead turtle nesting area in Turkey (Türkozan 2000). From the literature, we identified a total of six adult loggerhead turtles (three male and three female) that migrated into Amvrakikos Gulf; all from the Greek nesting area of Zakynthos (Zbinden et al. 2011; Schofield et al. 2013).

### Links from genetics

**Fig. 2:**
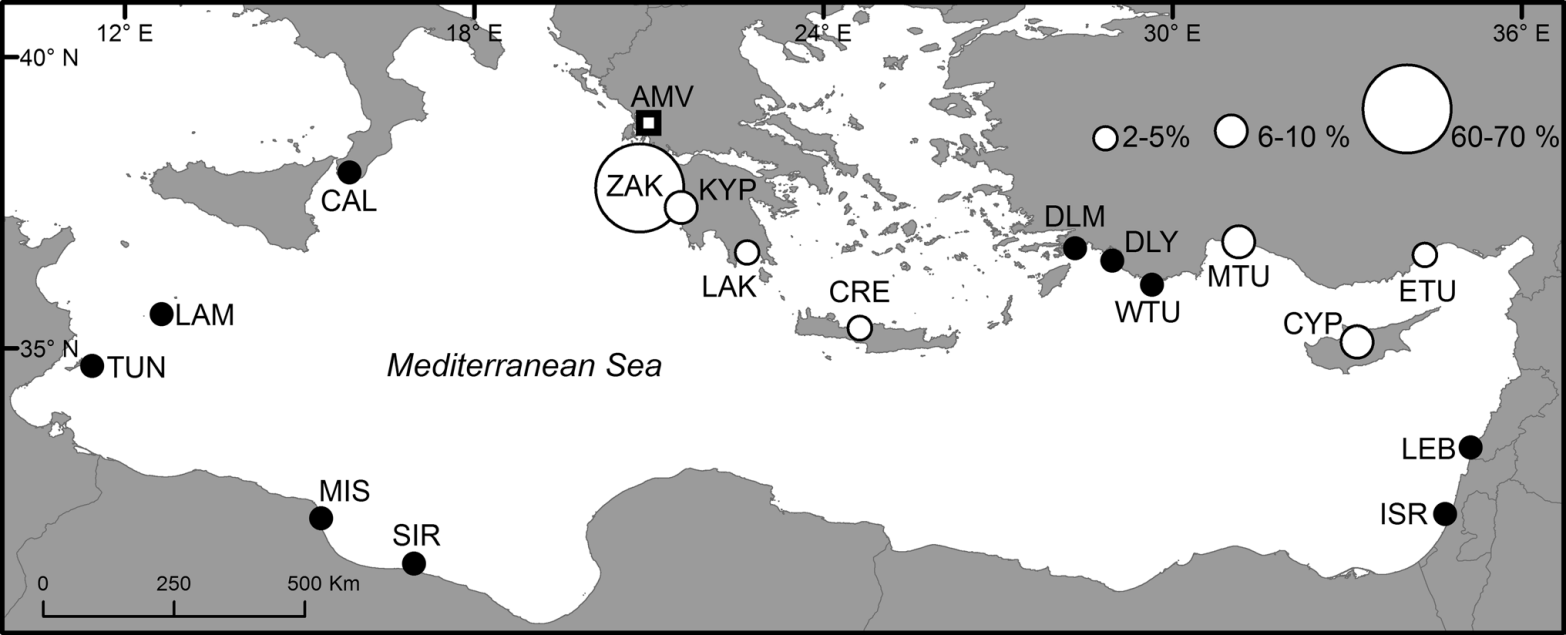
Proportional contribution of Mediterranean nesting units to the foraging population in Amvrakikos Gulf using genetic markers. *Circles* Mediterranean nesting units that have been defined for mitochondrial haplotype frequencies in previous studies (Garofalo et al. 2009, Yilmaz et al. 2011, Clusa et al. 2013, Carreras et al. 2014) and considered for Mixed Stock Analysis of Amvrakikos (AMV) samples. *Open circles* indicate a nesting unit contribution of >2% to the Amvrakikos Gulf aggregation, with size denoting percentage contribution. Greece (*ZAK* Laganas Bay—Zakynthos Island, *KYP* southern Kyparissia Bay, *LAK* Lakonikos Bay, *CRE* Rethymno—Crete Island), Turkey (*DLM* Dalaman, *DLY* Dalyan, *WTU* western Turkey, *MTU* mid Turkey, *ETU* eastern Turkey), Cyprus (*CYP*), Lebanon (*LEB*), Israel (*ISR*), Libya (*SIR* Sirte, *MIS* Misurata), Tunisia (*TUN* Kuriat Islands), Italy (*LAM* Lampedusa Island, *CAL* Calabria)

**Fig. 3:**
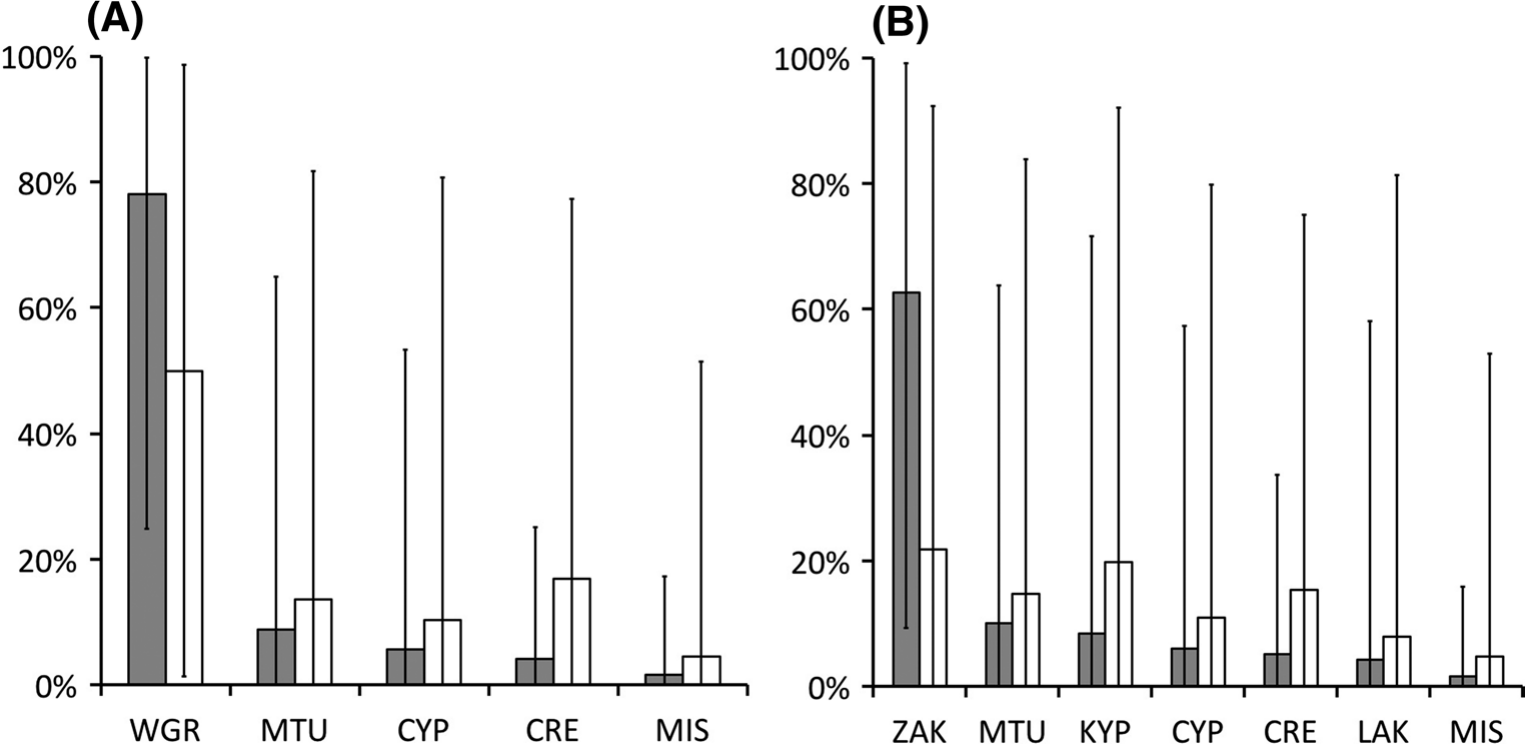
Mixed stock analysis (MSA) of loggerhead turtles from Amvrakikos Gulf foraging area, Greece (*grey bars*), and comparison with similar analysis on turtles from Drini Bay foraging area in Albania (*white bars*) (Yilmaz et al. 2012). **a** MSA using defined breeding units (see Fig. 2). **b** MSA with nesting areas in the western Greece unit (WGR) incorporated individually (ZAK, KYP, LAK) to identify likely source nesting beaches. See Fig. 2 for locations and abbreviations. Charts are truncated at the 2% contribution level; data for all areas are available in Supplemental Table S1. *Error bars* show 95% confidence interval range

A total of four haplotypes were recorded from Amvrakikos Gulf turtles (Table 2): the widespread haplotype CC-A2.1 (Genebank EU179445; Shamblin et al. 2012), the Crete haplotype CC-A2.8 (Genebank FM200217; Garofalo 2010), and the Greek haplotypes CC-A6.1 (Genebank JQ350705; Yilmaz et al. 2012; Clusa et al. 2013) and CC-A32.1 (Genebank JF837822; Clusa et al. 2013). A preliminary mixed stock analysis including all Atlantic and Mediterranean nesting areas showed that collectively the Atlantic nesting areas pooled was less than 1% of the Amvrakikos sea turtles (data not shown). A second analysis was performed excluding all these Atlantic nesting areas, as previous studies suggested removing from the analysis those nesting areas whose contribution was biologically unrealistic and considering that no Atlantic exclusive haplotypes was found within our samples (Engstrom et al. 2002; Godley et al. 2010). This second mixed stock analysis showed that most of the Amvrakikos turtles (78%) originated from western Greece (Fig. 3; Supplemental Table S1), although notable contributions from Crete, Mid Turkey and Cyprus were also inferred (Figs. 2 and 3; Supplemental Table S1). Further analysis was undertaken for a fine scale assignation, considering western Greece nesting beaches (Zakynthos, southern Kyparissia and Lakonikos Bay) separately, justified by Zakynthos presents an exclusive haplotype. Zakynthos was the origin of most (63%) of western Greek turtles located in Amvrakikos, followed by southern Kyparissia (8%) and Lakonikos nesting areas (4%) (Fig. 3; Supplemental Table S1).

**Table 2:** Haplotype frequencies from Amvrakikos Gulf (Greece) and Drini Bay (Albania) feeding grounds and the Mediterranean nesting areas used for the mixed stock analysis

	CC-A2.1	CC-A2.8	CC-A2.9	CC-A3.1	CC-A3.2	CC-A6.1	CC-A10.4	Cc-A13.1	CC-A20.1	CC-A26.1	CC-A29.1	CC-A31.1	CC-A32.1	CC-A43.1	CC-A50.1	CC-A52.1	CC-A53.1	CC-A65.1	CC-A68.1	*n*	Ref.
Amvrakikos Gulf, Greece—AMV	88	1				4							2							95	A
Drini Bay, ALBANIA	37	1				1	1													40	G
ITALY																					
Calabria—CAL	22								14			2								38	B
LIBYA																					
Misurata—MIS	12		1	1																14	C
Sirte—SIR	16		12	2						4									1	35	C
GREECE																					
Zakynthos—ZAK	16					2							1							19	D
Kyparissia—KYP	33					2						1								36	E
Lakonikos—LAK	18					1														19	D
Crete—CRE	16	4																		20	D
CYPRUS																					
CYP	44														1					45	D
TURKEY																					
Dalyan—DLY	25			15																40	F
Dalaman—DLM	5			15																20	F
Western Turkey—WTU	60			16																76	F
Mid Turkey—MTU	46							1												47	F
Eastern Turkey—ETU	60			8	1									1		1	1			72	F
LEBANON																					
LEB	17			2																19	D
ISRAEL																					
ISR	15		2								2									19	D

A—Present study; B—Garofalo et al. 2009, C—Saied et al. 2012; D—Clusa et al. 2013; E—Carreras et al. 2014; F—Yilmaz et al. 2011; G—Yilmaz et al. 2012. WTU, MTU and ETU are groups of nesting beaches as defined in Yilmaz et al. (2011). ZAK, KYP and LAK were grouped as WGR for some analysis, as per Carreras et al. (2014)

The turtles from Drini Bay, Albania (CCL Mean ± SD; 68.8 ± 10.3 cm, *n* = 40) were similar in size, and therefore life-stages, to those sampled from Amvrakikos Gulf (69.9 ± 8.3 cm, *n* = 94). MSAs using the Albanian haplotype frequencies presented by Yilmaz et al. (2012) indicated the majority (50%) of turtles present originated from western Greece, with larger contributions from the other same three other nesting units (Crete, Mid Turkey and Cyprus; Fig. 3) and if western Greece beaches were included separately in the MSA, Zakynthos was again shown to contribute the single largest proportion of turtles (22%), although far less dominant when compared to Amvrakikos (Fig. 3; Supplemental Table S1).

## Discussion

Our results, from three differing research methods, indicate that turtles converge to use the same foraging areas from several breeding areas within 600 km distance. Thus further highlighting the utility of complementary studies in determining levels of reproductive and developmental isolation for widespread populations (Godley et al. 2003; Stewart et al. 2013).

### Links from flipper tagging and satellite telemetry

Mark-recapture revealed links with proximate nesting areas in Greece, through cross-sighting of adult females tagged at a nesting beach and Amvrakikos Gulf. These breeding sites represent some of the largest populations in the Mediterranean (i.e. Laganas Bay, Zakynthos and southern Kyparissia Bay, the Peloponnese; Margaritoulis et al. 2003). The six turtles tracked from Zakynthos into Amvrakikos (Zbinden et al. 2011; Schofield et al. 2013) represent 9% of the total number of turtles tracked from that location over an eight-year period. A single turtle tracked from Kefalonia (Hays et al. 1991) and another from Rethymno on Crete (Margaritoulis and Rees 2011) represent the only published tracks from other Greek nesting areas and consequently the biased effort precludes using satellite tracking data for direct comparison of the proportion of turtles from different breeding areas utilising Amvrakikos. Although no turtles tagged outside Greece have been recorded within Amvrakikos Gulf, tag recaptures from nesting females in Turkey (*n* = 1) and Cyprus (*n* = 1) have been recorded in the Adriatic Sea (Lazar et al. 2004), which necessitates passing the entrance to Amvrakikos Gulf.

A modelling approach indicates hatchling turtles from Cyprus would disperse to the northern Ionian Sea region (Casale and Mariani 2014) and stable isotope analysis of breeding female loggerhead turtles from across the eastern Mediterranean indicates that a notable proportion from each location forages in the northern Ionian Sea or Adriatic Sea (Clusa et al. 2014). It is therefore intriguing that despite a long-term tracking programme on Cyprus (Snape et al. 2016), loggerhead turtles from there are yet to demonstrate northern Ionian/Adriatic directed migrations and the single flipper-tag recapture (Lazar et al. 2004) remains the only direct evidence of migration into the region.

### Life stage and sex bias

All long-distance tag recaptures and satellite tracking results to date that relate to Amvrakikos Gulf have involved adult individuals and mainly females (13 females from 16 individuals), and yet the majority of the turtles found in Amvrakikos are juveniles, sub-adults and male (231 from 278 turtles; Rees et al. 2013). This bias in life stage representation may be due to different habitat preferences of males and females, as the study only takes place in a small part of the Gulf, but more likely relates to the opportunity for observation and tagging outside the foraging area. Nesting females are the most commonly encountered life stage and sex due to their predictable appearance on traditional nesting beaches and as such are the subject of the majority of flipper tagging and satellite tracking studies (reviewed in Godley et al. 2008; Jeffers and Godley 2016).

Tagging and tracking studies of male and juvenile loggerhead turtles in the eastern Mediterranean, have sourced animals from fisheries bycatch and/or rehabilitation facilities in three main locations, (1) the Adriatic Sea (White et al. 2011; Casale et al. 2012a), (2) southern Italian waters (Bentivegna 2002; Casale et al. 2007), and (3) waters of the African continental shelf off Tunisia and Libya (Hochscheid et al. 2010; Casale et al. 2012b, 2013), with some exceptions (Schofield et al. 2010; present study). There is a complete lack of published information from further east (Turkey, Cyprus, the Levant coast and Egypt). No individuals from the African continental shelf region have yet been recorded in the northern Ionian or Adriatic seas whereas there have been several recorded from the southern Italian waters. This is highly suggestive of geographical developmental segregation, with a large latitudinal component and the key driver stimulating any north–south movement in adulthood might be the need to undertake reproductive migrations (Margaritoulis et al. 2003; Broderick et al. 2007; Zbinden et al. 2011; Casale et al. 2013; Schofield et al. 2013).

### Links from genetics

As with other sea turtle studies (Lahanas et al. 1998; Roberts et al. 2005; Reece et al. 2006; Prosdocimi et al. 2012), we have successfully used genetic markers to estimate breeding stock contributions to the Amvrakikos Gulf assemblage. Our results suggest the majority of the turtles in Amvrakikos are of Greek origin, supporting the assertion that loggerhead turtles locate their maturation and adult life stages closer to their nesting areas than would be expected from random settling after pelagic dispersal (Bowen et al. 2004; Reece et al. 2006; Casale et al. 2007; Pajuelo et al. 2012; Garofalo et al. 2013). Lack of fidelity to breeding areas witnessed in some individuals nesting in Greece (Margaritoulis 1998; present study) would support the western Greece grouping of breeding populations (Carreras et al. 2007; Clusa et al. 2013; Casale and Mariani 2014). However, when analysed in detail, the majority of turtles in Amvrakikos Gulf are indicated to originate from a single nesting area. Zakynthos is calculated to contribute 63% of the turtles characterised in genetic analysis, which is a similar level of contribution derived from flipper and satellite tracking studies incorporating only adults (see above).

Predominance of Greek and specifically Zakynthos contributions to foraging loggerhead turtles sampled in Drini Bay reinforce this assumption of proximal settlement to natal breeding area, with dispersal linked to original, passive movements in currents experienced by hatchlings (Hays et al. 2010a). Again these genetic data are further supported by two adult females tagged in Zakynthos being recaptured in Drini Bay (White et al. 2011) and no other tag recaptures linking the area to other nesting grounds.

Genetic analyses indicate Amvrakikos Gulf and Drini Bay are of international relevance, as turtles foraging in this region are the most productive (Cardona et al. 2014). Low contributions to both assemblages from Turkish and Cypriot nesting areas and tag returns from the Adriatic (Lazar et al. 2004) confirm that migrations to this region from these locations occur at low levels. However, mixed stock analysis using mtDNA relies on breeding isolation and characterisation of individual nesting areas. Despite the greater resolution the increased-length mtDNA sequences have provided over the previously used shorter sequences (Shamblin et al. 2012; Clusa et al. 2013), the Mediterranean loggerhead turtle populations remain linked through the common CCA2.1 haplotype that accounts for 80% or more of the samples in nine of the 15 nesting areas assessed (Table 2) and reaches over 95% in three (Lakonikos Bay in Greece, Cyprus & Mid Turkey). Additionally, not all haplotypes that have been described in the Mediterranean can be incorporated in MSA (as smaller datasets are dropped to avoid pseudoreplication, see Carreras et al. 2014) thus weakening their potential resolution.

No haplotypes apparently endemic to breeding areas outside Greece were obtained in our current analysis, thus when Greek endemic and common haplotypes are combined in a MSA with population size as a prior the results are likely to indicate contributions from larger non-Greek nesting areas. The Amvrakikos Gulf MSA results display wide confidence limits around average contribution levels reported here (Fig. 3; Supplemental Table S1); consequently, the smaller contributions of populations outside of Greece, namely Turkey, Cyprus and Libya, should be accepted with caution, until better supported by other lines of evidence (such as capture-mark-recapture investigations and satellite tracking).

Furthermore, mtDNA only assesses matrilineal reproductive isolation. Some levels of male mediated gene flow, demonstrated by lower differentiation in nuclear DNA compared to mtDNA have been recorded in the Mediterranean (Schroth et al. 1996; Carreras et al. 2007; Yilmaz et al. 2012). Nuclear DNA revealed that Crete and Cyprus nesting areas play pivotal roles in homogenising Mediterranean stocks (Carreras et al. 2007). However, these islands might not be exclusive locations for potential genetic mixing. Loggerhead turtle mating has been reported in Amvrakikos Gulf (Teneketzis et al. 2003) and hence the potential for individuals that hatched on beaches separated by thousands of kilometres to interbreed in distant foraging areas. This genetic mixing may increase the genetic variability of loggerhead turtle nesting aggregations, making the smaller aggregations more robust against inbreeding depression (Bell et al. 2009). However, natal philopatry in females means that recovery in nesting levels for compromised or near extirpated populations requires recruitment of individuals with ancestral origins at the affected sites (Carreras et al. 2007; Watanabe et al. 2011). It is therefore important to protect established nesting areas as well as foraging hot-spots to properly conserve the extant genetic diversity within the meta-population. Conservation measures adopted within Amvrakikos Gulf, which hosts year-round, regionally important numbers of loggerhead turtles (Rees et al. 2013) will help preserve both the larger breeding populations of Greece, including that of Zakynthos and the distant, more depleted nesting areas in Turkey, Cyprus and Libya.

### Theory to explain male-biased sex ratio

An unexplained, high proportion of male turtles has been reported in Amvrakikos Gulf, with male-biased sex ratios in the largest size classes (Rees et al. 2013). This is contrary to the female-biased sex ratios produced at most Mediterranean nesting areas (Witt et al. 2010a with additional data in Uçar et al. 2012). This male bias is likely a consequence of several factors.

Approximately 60% of turtles in Amvrakikos Gulf originate from the large breeding aggregation at the relatively nearby Zakynthos. The sex ratio of hatchlings from that Island is vary variable, with a long-term average of around 50% male hatchling production (Compare Zbinden et al. 2007 with Katselidis et al. 2012). Additionally, fieldwork in the Gulf has been carried during the Mediterranean turtle nesting season, hence a cohort of adult females would be absent from the Gulf to breed, but the males would have already departed from the nesting areas to return to foraging areas such as Amvrakikos Gulf (Hays et al. 2010b). Furthermore, there is growing evidence that adult male turtles remain closer to the nesting areas than female conspecifics. This has been shown in loggerhead turtles (Arendt et al. 2012; Schofield et al. 2013) and hawksbill turtles *Eretmochelys imbricata* (Van Dam et al. 2008) and may facilitate more frequent remigration in males (Wright et al. 2012; Hays et al. 2014).

## Conclusions

Through the integration of flipper tagging, satellite tracking and genetic markers we have garnered a number of insights, both fundamental and applied. We advance understanding of factors affecting natal homing and location-specific variation in sex ratios in foraging grounds (see Pilcher et al. 2015). Congruence in data obtained from low- and high-tech methodologies validate genetic designations of statistical ambiguity, generating defensible results that may serve as a template for other foraging ground studies. To take this work further for the site in question would involve aerial surveying to better understand heterogeneity of habitat use (e.g. Seminoff et al. 2014) that can be followed up by focussed in-water work.

It is likely that advances in genetic marker resolution, in tandem with increased sample sizes and sampling from some of more sparsely nested areas, would reveal further structuring within foraging turtle aggregations. This could lead to more assured individual-level assignments regarding natal origins, in contrast to the few individuals that may currently be assigned through low-frequency endemic haplotypes. Additional methods of characterising individuals to a geographical area, such as use of biochemical markers like stable isotopes (Zbinden et al. 2011; Ceriani et al. 2012; Shimada et al. 2014), even in the absence of external markers, could enhance our understanding of a specific foraging area’s region-wide links to nesting sites through complementary sampling of individuals observed nesting.

Finally, data sharing through publication and collaboration produces enhanced datasets that can be subjected to more rigorous analysis from which more robust inferences drawn. These stepwise contributions of complementary insights into the life-histories of sea turtles may directly impact conservation measures, as management plans are revised in line with the latest ecological findings (Fujioka and Halpin 2014).

## Electronic supplementary material

Below is the link to the electronic supplementary material.

Supplementary material 1 (DOCX 92 kb)
