# Supplementary material for: Linking loggerhead locations: using multiple methods to determine the origin of sea turtles in feeding grounds
Source: Mar Biol. 2017 Jan 13;164(2):30. doi: 10.1007/s00227-016-3055-z (PMC5236075; doi:10.1007/s00227-016-3055-z)
Supplement: Supplementary file 1 — Supplementary material 1 (DOCX 92 kb) [file 227_2016_3055_MOESM1_ESM.docx]

**SUPPLEMENTAL MATERIAL**

**Table S1** Nesting area contributions to the Amvrakikos Gulf and Drini Bay foraging aggregations determined by mixed stock analysis. Greece (ZAK = Laganas Bay - Zakynthos Island, KYP = southern Kyparissia Bay, LAK = Lakonikos Bay, CRE = Rethymno - Crete Island; WGR is a genetically distinct unit (Carreras et al. 2014) comprising ZAK, KYP & LAK), Turkey (DLM = Dalaman, DLY = Dalyan, WTU = western Turkey, MTU = mid Turkey, ETU = eastern Turkey), Cyprus (CYP), Lebanon (LEB), Israel (ISR), Libya (SIR = Sirte, MIS = Misurata), Tunisia (TUN = Kuriat Islands), Italy (CAL = Calabria).

A) Grouped nesting areas

| **Amvrakikos Gulf** | | | | | |
| --- | --- | --- | --- | --- | --- |
| **STOCK** | **MEAN** | **SD** | **2.5%** | **MEDIAN** | **97.5%** |
| WGR | 0.7806 | 0.218 | 0.2483 | 0.8613 | 0.9984 |
| MTU | 0.088 | 0.1754 | 0 | 0.0016 | 0.6492 |
| CYP | 0.0562 | 0.1366 | 0 | 0.0002 | 0.5326 |
| CRE | 0.0411 | 0.0748 | 0 | 0.007 | 0.2515 |
| MIS | 0.0159 | 0.0483 | 0 | 0.0001 | 0.1732 |
| ETU | 0.0086 | 0.0282 | 0 | 0 | 0.085 |
| WTU | 0.0036 | 0.0149 | 0 | 0 | 0.04 |
| LEB | 0.0024 | 0.0166 | 0 | 0 | 0.0206 |
| SIR | 0.0013 | 0.0053 | 0 | 0 | 0.0144 |
| DLY | 0.0012 | 0.0064 | 0 | 0 | 0.0132 |
| ISR | 0.0006 | 0.0056 | 0 | 0 | 0.0038 |
| DLM | 0.0003 | 0.0024 | 0 | 0 | 0.0026 |
| CAL | 0.0001 | 0.0019 | 0 | 0 | 0 |

| **Drini Bay** | | | | | |
| --- | --- | --- | --- | --- | --- |
| **STOCK** | **MEAN** | **SD** | **2.5%** | **MEDIAN** | **97.5%** |
| WGR | 0.499 | 0.3053 | 0.0136 | 0.4944 | 0.9863 |
| MTU | 0.1366 | 0.2343 | 0 | 0.0049 | 0.817 |
| CYP | 0.104 | 0.2163 | 0 | 0.0007 | 0.8068 |
| CRE | 0.1689 | 0.2134 | 0 | 0.0815 | 0.7726 |
| MIS | 0.0452 | 0.13 | 0 | 0.0003 | 0.5144 |
| ETU | 0.0174 | 0.0532 | 0 | 0.0001 | 0.1776 |
| WTU | 0.0131 | 0.0521 | 0 | 0 | 0.1429 |
| LEB | 0.006 | 0.0445 | 0 | 0 | 0.0388 |
| SIR | 0.0035 | 0.0147 | 0 | 0 | 0.0383 |
| DLY | 0.0032 | 0.0178 | 0 | 0 | 0.0346 |
| ISR | 0.0022 | 0.0186 | 0 | 0 | 0.0138 |
| DLM | 0.0005 | 0.0038 | 0 | 0 | 0.0048 |
| CAL | 0.0004 | 0.0064 | 0 | 0 | 0 |

B) Nesting areas, with the western Greece group (ZAK, KYP, LAK) treated as individual areas

| **Amvrakikos Gulf** | | | | | |
| --- | --- | --- | --- | --- | --- |
| **STOCK** | **MEAN** | **SD** | **2.5%** | **MEDIAN** | **97.5%** |
| ZAK | 0.6266 | 0.2799 | 0.0936 | 0.675 | 0.9912 |
| MTU | 0.1012 | 0.1792 | 0 | 0.0037 | 0.6378 |
| KYP | 0.0843 | 0.1851 | 0 | 0.0011 | 0.7156 |
| CYP | 0.0608 | 0.1471 | 0 | 0.0002 | 0.5738 |
| CRE | 0.0521 | 0.0941 | 0 | 0.0139 | 0.3372 |
| LAK | 0.0431 | 0.1395 | 0 | 0 | 0.5816 |
| MIS | 0.0167 | 0.0559 | 0 | 0.0001 | 0.159 |
| ETU | 0.0072 | 0.0241 | 0 | 0 | 0.071 |
| WTU | 0.0034 | 0.0137 | 0 | 0 | 0.0391 |
| LEB | 0.0014 | 0.0106 | 0 | 0 | 0.0105 |
| SIR | 0.0012 | 0.0052 | 0 | 0 | 0.0139 |
| DLY | 0.0011 | 0.0064 | 0 | 0 | 0.0117 |
| ISR | 0.0006 | 0.0065 | 0 | 0 | 0.0028 |
| DLM | 0.0003 | 0.0022 | 0 | 0 | 0.002 |
| CAL | 0.0001 | 0.0014 | 0 | 0 | 0 |

| **Drini Bay** | | | | | |
| --- | --- | --- | --- | --- | --- |
| **STOCK** | **MEAN** | **SD** | **2.5%** | **MEDIAN** | **97.5%** |
| ZAK | 0.2187 | 0.278 | 0 | 0.0855 | 0.9229 |
| MTU | 0.1482 | 0.2513 | 0 | 0.0045 | 0.8387 |
| KYP | 0.1979 | 0.2843 | 0 | 0.0294 | 0.9207 |
| CYP | 0.1099 | 0.2184 | 0 | 0.0011 | 0.7988 |
| CRE | 0.1538 | 0.2024 | 0 | 0.0717 | 0.7504 |
| LAK | 0.0793 | 0.2072 | 0 | 0 | 0.8137 |
| MIS | 0.0477 | 0.1333 | 0 | 0.0003 | 0.5296 |
| ETU | 0.0175 | 0.0545 | 0 | 0.0001 | 0.1836 |
| WTU | 0.0107 | 0.0474 | 0 | 0 | 0.111 |
| LEB | 0.007 | 0.0545 | 0 | 0 | 0.0363 |
| SIR | 0.0032 | 0.0135 | 0 | 0 | 0.0355 |
| DLY | 0.0033 | 0.0184 | 0 | 0 | 0.0366 |
| ISR | 0.002 | 0.0175 | 0 | 0 | 0.0089 |
| DLM | 0.0006 | 0.0047 | 0 | 0 | 0.0052 |
| CAL | 0.0002 | 0.0026 | 0 | 0 | 0 |
